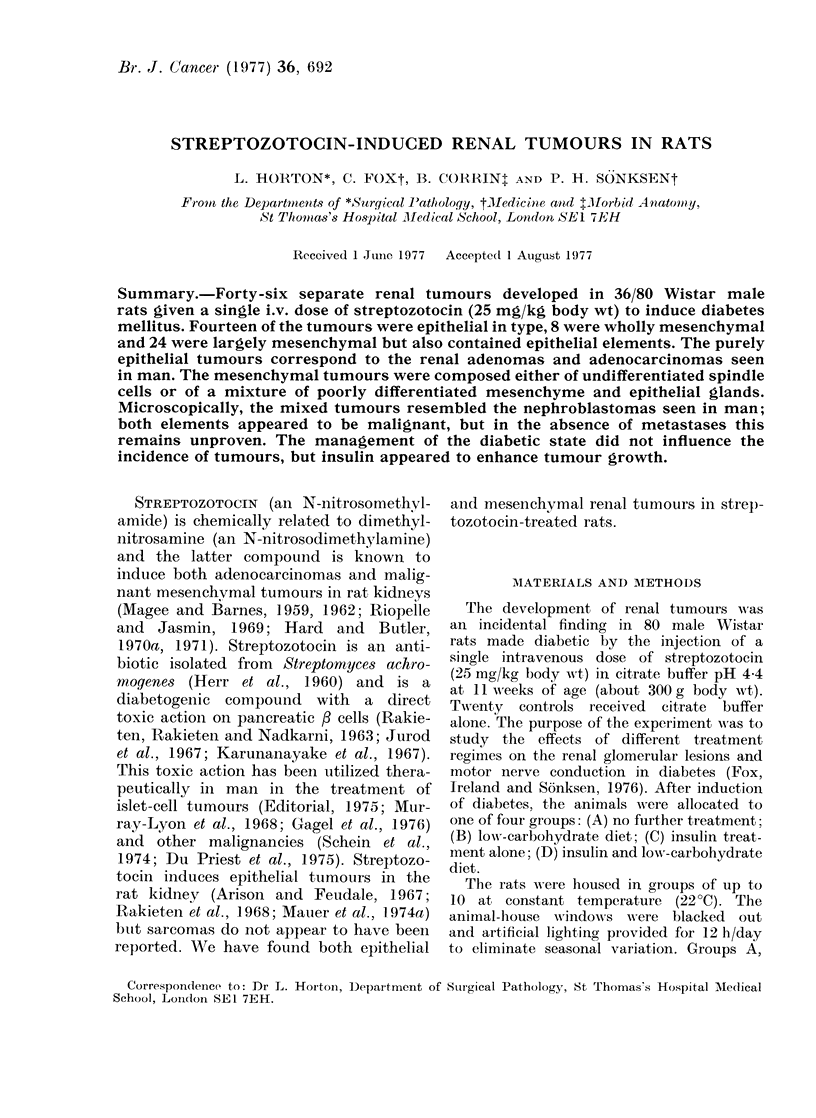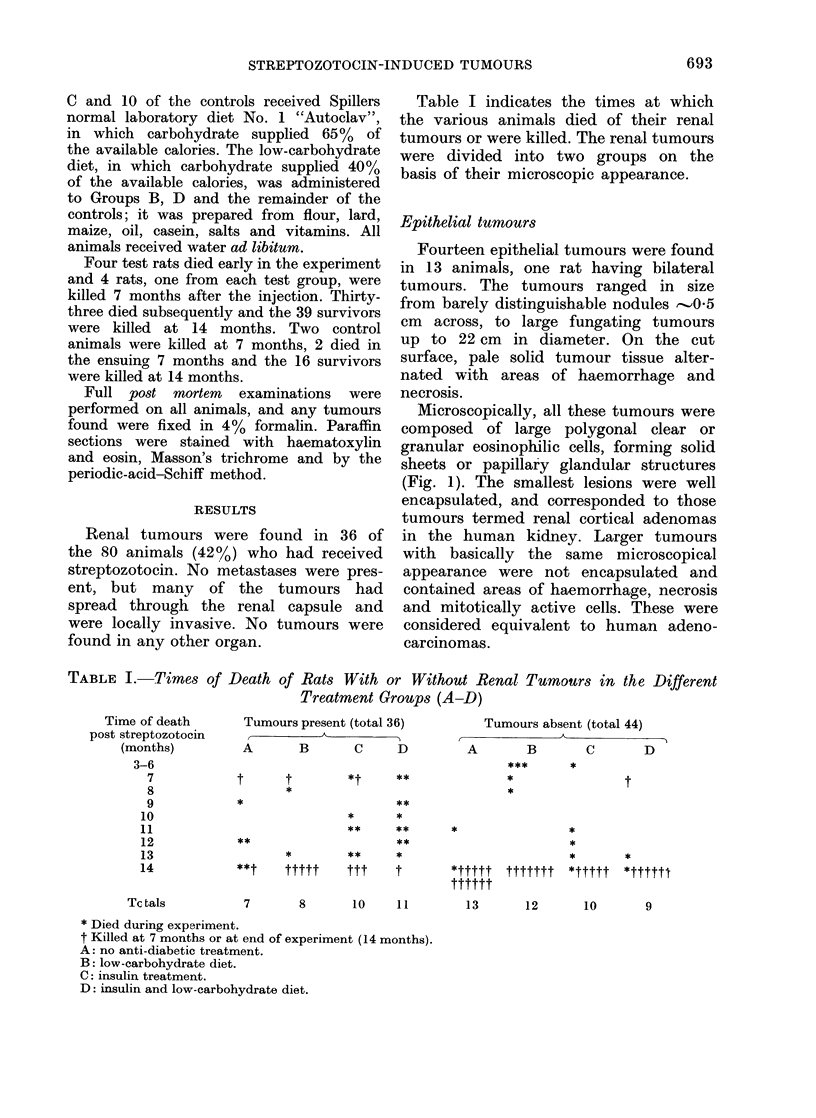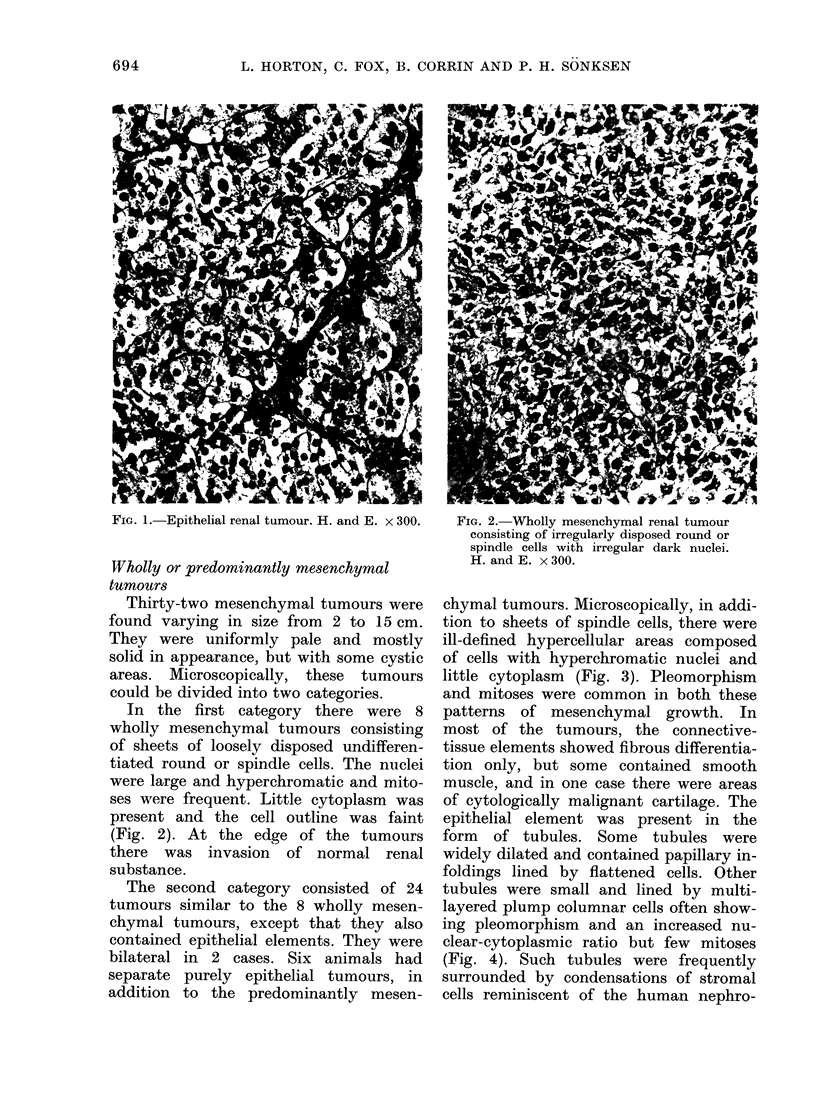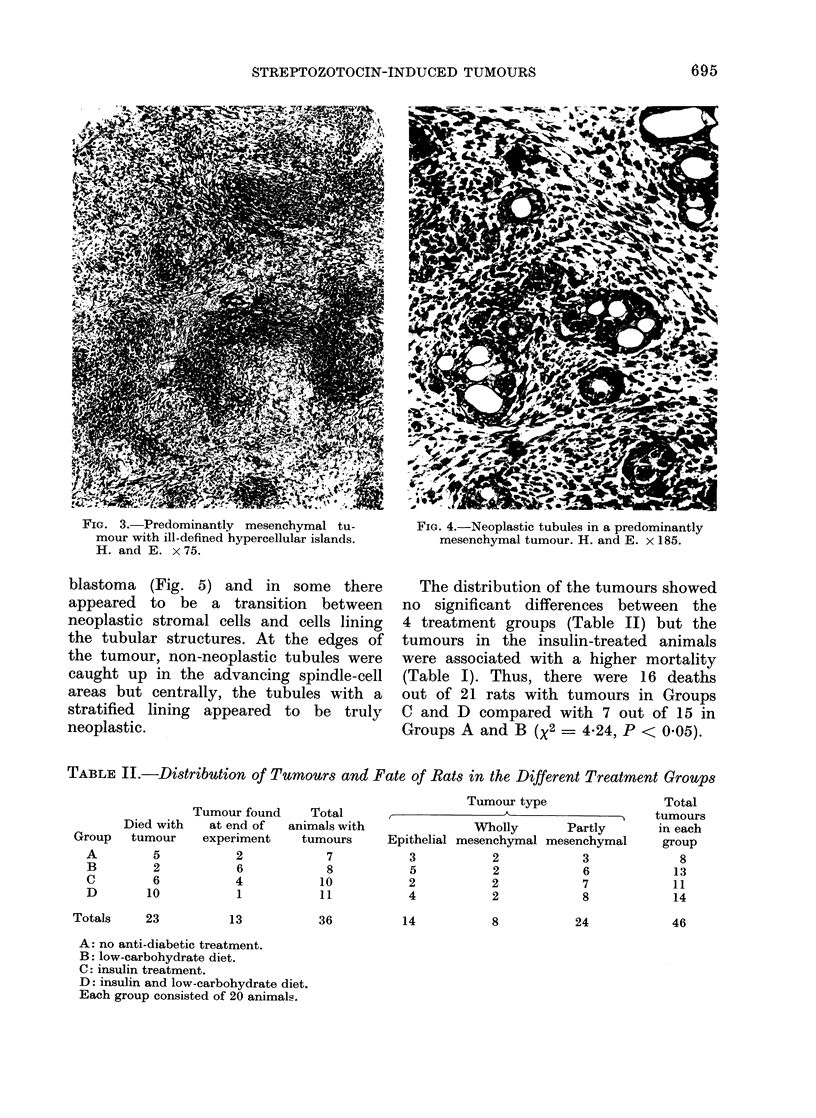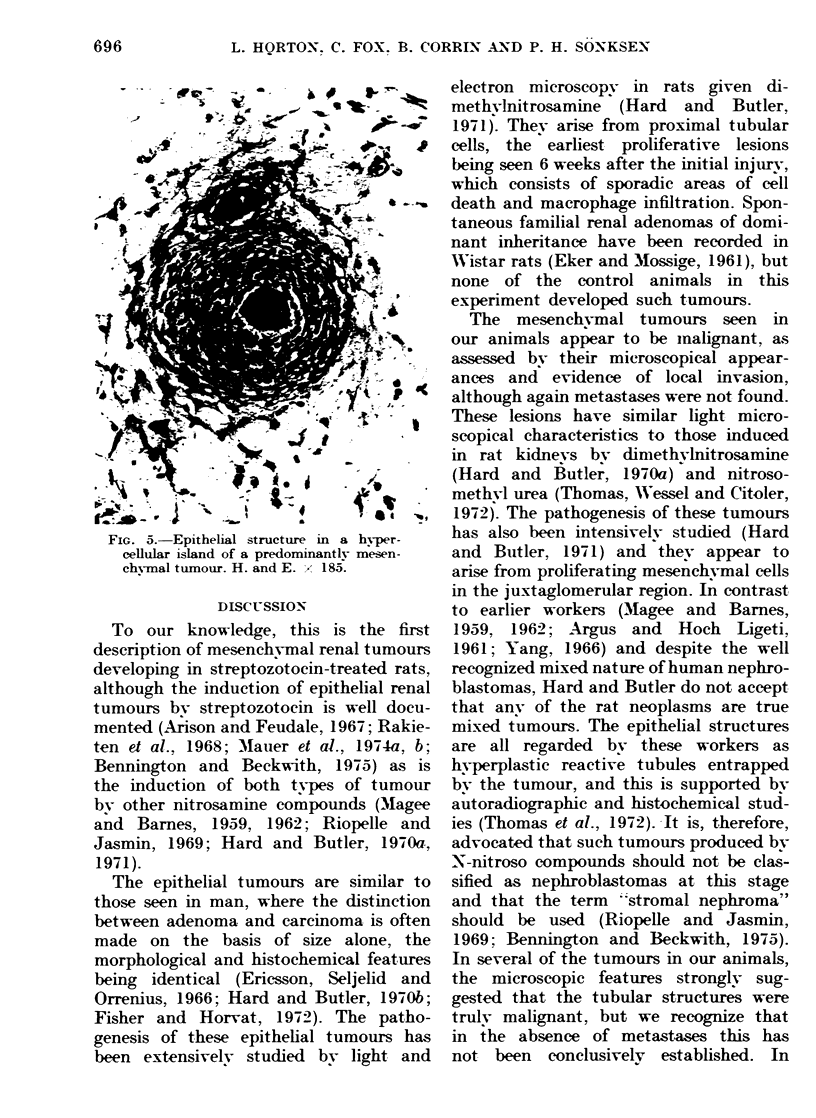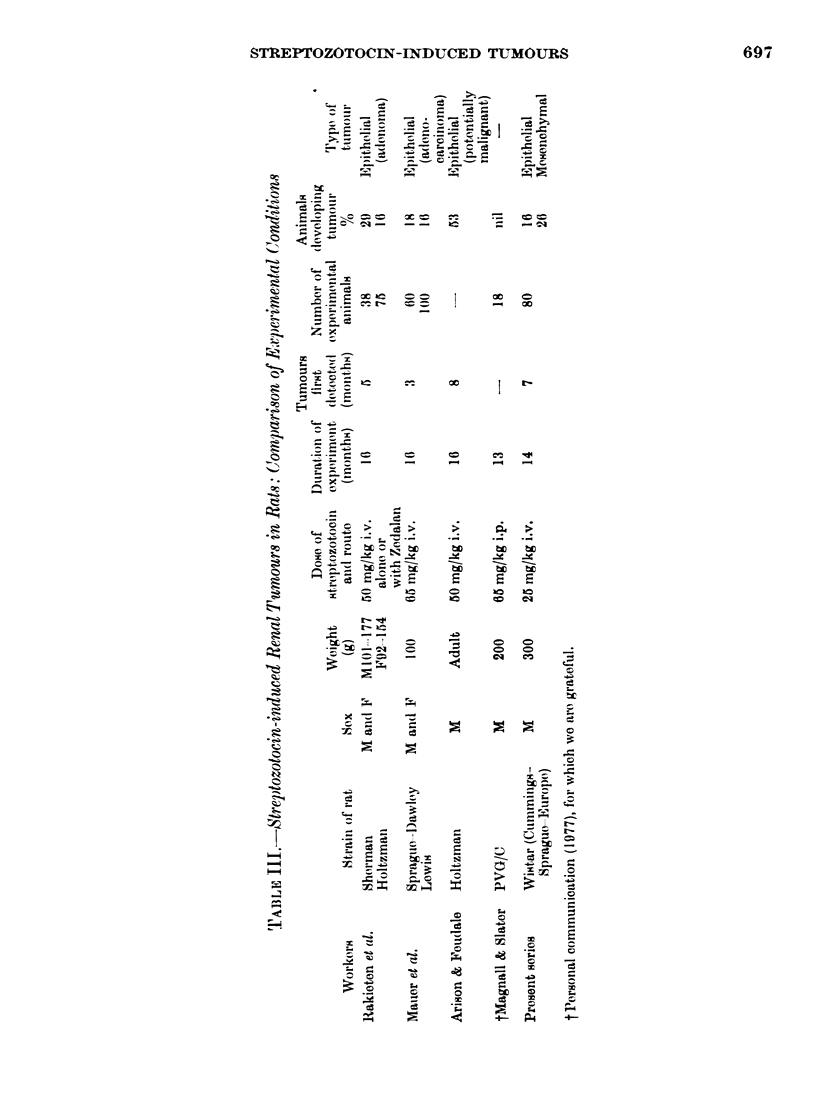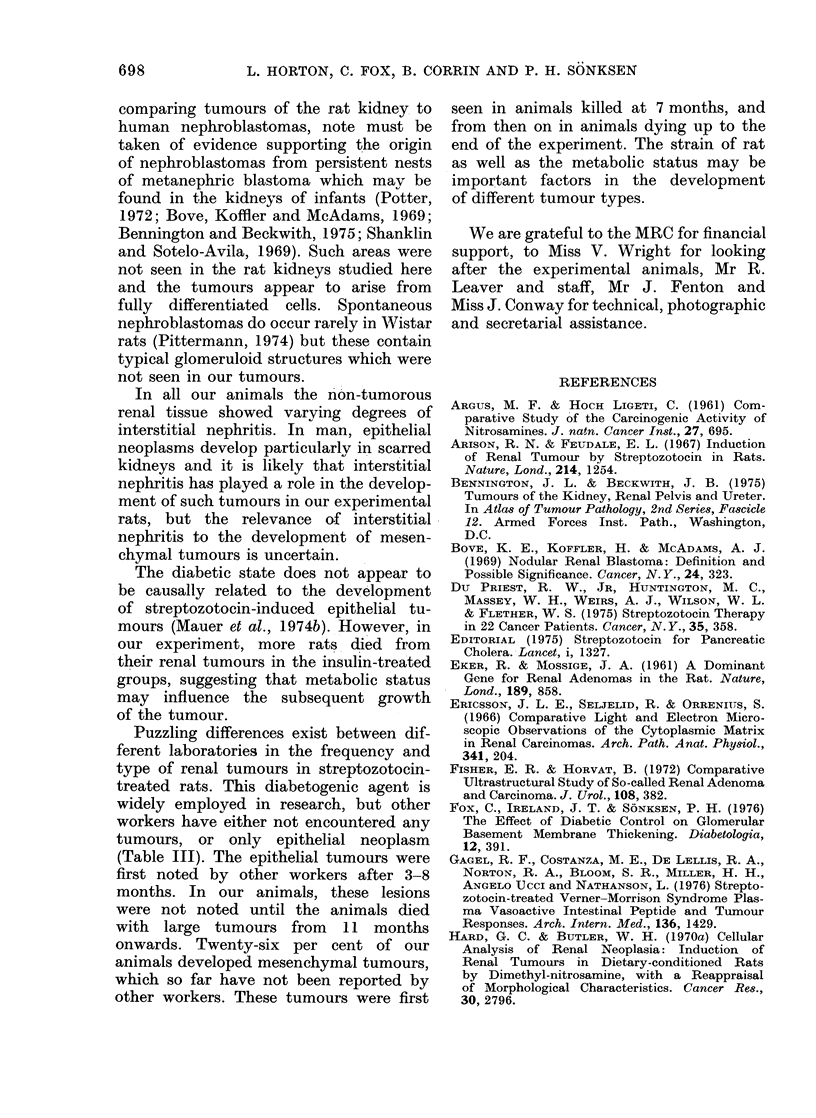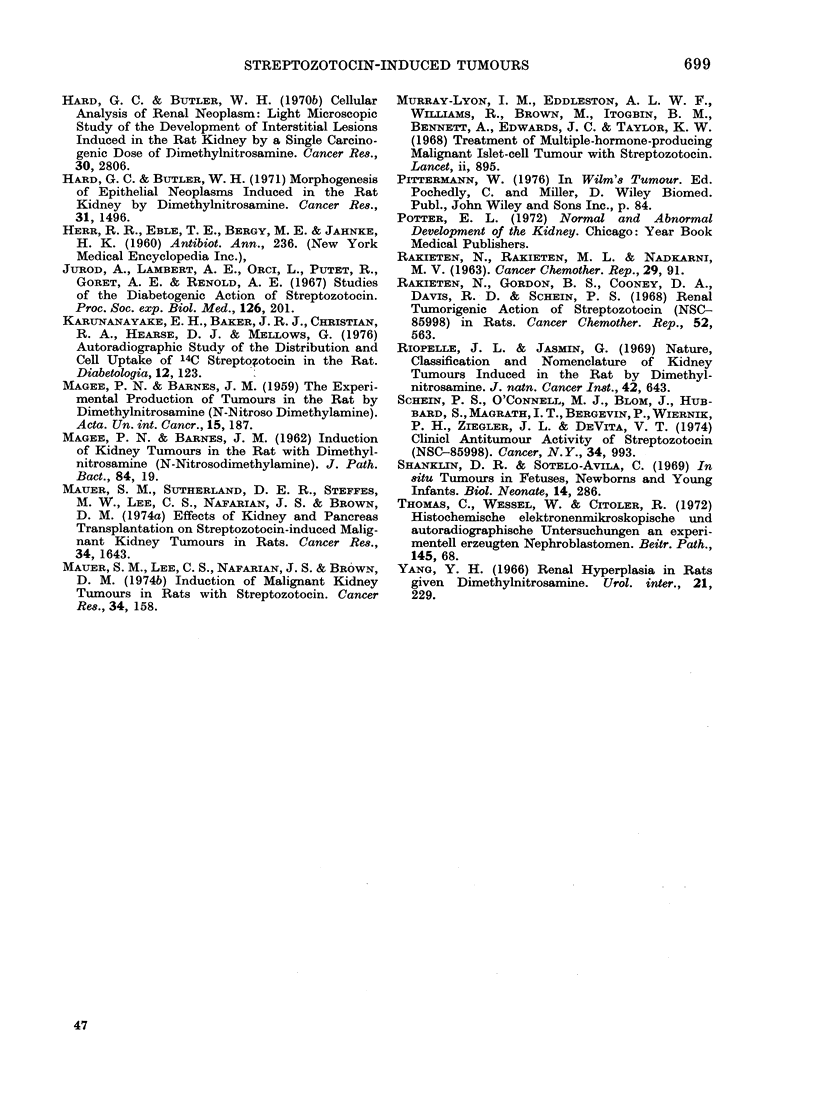# Streptozotocin-induced renal tumours in rats.

**DOI:** 10.1038/bjc.1977.251

**Published:** 1977-12

**Authors:** L. Horton, C. Fox, B. Corrin, P. H. Sönksen

## Abstract

**Images:**


Br. J. Cancer (1977) 36, 692

### STREPTOZOTOCIN-INDUCED RENAL TUMOURS IN RATS

L. HORTON*, C. FOX†, B. CORRIN‡ AND P. H. SÖNKSEN†

*From the Departments of *Surgical Pathology, †Medicine and ‡Morbid Anatomy, St Thomas's Hospital Medical School, London SE1 7EH*

Received 1 June 1977 Accepted 1 August 1977

**Summary.—Forty-six separate renal tumours developed in 36/80 Wistar male rats given a single i.v. dose of streptozotocin (25 mg/kg body wt) to induce diabetes mellitus. Fourteen of the tumours were epithelial in type, 8 were wholly mesenchymal and 24 were largely mesenchymal but also contained epithelial elements. The purely epithelial tumours correspond to the renal adenomas and adenocarcinomas seen in man. The mesenchymal tumours were composed either of undifferentiated spindle cells or of a mixture of poorly differentiated mesenchyme and epithelial glands. Microscopically, the mixed tumours resembled the nephroblastomas seen in man; both elements appeared to be malignant, but in the absence of metastases this remains unproven. The management of the diabetic state did not influence the incidence of tumours, but insulin appeared to enhance tumour growth.**

STREPTOZOTOCIN (an N-nitrosomethyl-amide) is chemically related to dimethyl-nitrosamine (an N-nitrosodimethylamine) and the latter compound is known to induce both adenocarcinomas and malignant mesenchymal tumours in rat kidneys (Magee and Barnes, 1959, 1962; Riopelle and Jasmin, 1969; Hard and Butler, 1970*a*, 1971). Streptozotocin is an antibiotic isolated from *Streptomyces achromogenes* (Herr *et al.*, 1960) and is a diabetogenic compound with a direct toxic action on pancreatic $\beta$ cells (Rakieten, Rakieten and Nadkarni, 1963; Jurod *et al.*, 1967; Karunanayake *et al.*, 1967). This toxic action has been utilized therapeutically in man in the treatment of islet-cell tumours (Editorial, 1975; Murray-Lyon *et al.*, 1968; Gagel *et al.*, 1976) and other malignancies (Schein *et al.*, 1974; Du Priest *et al.*, 1975). Streptozotocin induces epithelial tumours in the rat kidney (Arison and Feudale, 1967; Rakieten *et al.*, 1968; Mauer *et al.*, 1974*a*) but sarcomas do not appear to have been reported. We have found both epithelial and mesenchymal renal tumours in streptozotocin-treated rats.

### MATERIALS AND METHODS

The development of renal tumours was an incidental finding in 80 male Wistar rats made diabetic by the injection of a single intravenous dose of streptozotocin (25 mg/kg body wt) in citrate buffer pH 4·4 at 11 weeks of age (about 300 g body wt). Twenty controls received citrate buffer alone. The purpose of the experiment was to study the effects of different treatment regimes on the renal glomerular lesions and motor nerve conduction in diabetes (Fox, Ireland and Sönksen, 1976). After induction of diabetes, the animals were allocated to one of four groups: (A) no further treatment; (B) low-carbohydrate diet; (C) insulin treatment alone; (D) insulin and low-carbohydrate diet.

The rats were housed in groups of up to 10 at constant temperature (22°C). The animal-house windows were blacked out and artificial lighting provided for 12 h/day to eliminate seasonal variation. Groups A,

Correspondence to: Dr L. Horton, Department of Surgical Pathology, St Thomas's Hospital Medical School, London SE1 7EH.

C and 10 of the controls received Spillers normal laboratory diet No. 1 "Autoclav", in which carbohydrate supplied 65% of the available calories. The low-carbohydrate diet, in which carbohydrate supplied 40% of the available calories, was administered to Groups B, D and the remainder of the controls; it was prepared from flour, lard, maize, oil, casein, salts and vitamins. All animals received water *ad libitum*.

Four test rats died early in the experiment and 4 rats, one from each test group, were killed 7 months after the injection. Thirty-three died subsequently and the 39 survivors were killed at 14 months. Two control animals were killed at 7 months, 2 died in the ensuing 7 months and the 16 survivors were killed at 14 months.

Full *post mortem* examinations were performed on all animals, and any tumours found were fixed in 4% formalin. Paraffin sections were stained with haematoxylin and eosin, Masson's trichrome and by the periodic-acid–Schiff method.

### RESULTS

Renal tumours were found in 36 of the 80 animals (42%) who had received streptozotocin. No metastases were present, but many of the tumours had spread through the renal capsule and were locally invasive. No tumours were found in any other organ.

Table I indicates the times at which the various animals died of their renal tumours or were killed. The renal tumours were divided into two groups on the basis of their microscopic appearance.

### *Epithelial tumours*

Fourteen epithelial tumours were found in 13 animals, one rat having bilateral tumours. The tumours ranged in size from barely distinguishable nodules ~0·5 cm across, to large fungating tumours up to 22 cm in diameter. On the cut surface, pale solid tumour tissue alternated with areas of haemorrhage and necrosis.

Microscopically, all these tumours were composed of large polygonal clear or granular eosinophilic cells, forming solid sheets or papillary glandular structures (Fig. 1). The smallest lesions were well encapsulated, and corresponded to those tumours termed renal cortical adenomas in the human kidney. Larger tumours with basically the same microscopical appearance were not encapsulated and contained areas of haemorrhage, necrosis and mitotically active cells. These were considered equivalent to human adenocarcinomas.

TABLE I.—*Times of Death of Rats With or Without Renal Tumours in the Different Treatment Groups (A–D)*

| Time of death post streptozotocin (months) | Tumours present (total 36) | | | | Tumours absent (total 44) | | | |
|---|---|---|---|---|---|---|---|---|
| | A | B | C | D | A | B | C | D |
| 3–6 | | | | | | *** | * | |
| 7 | † | † | *† | ** | | * | | † |
| 8 | | * | | | | * | | |
| 9 | * | | | ** | | | | |
| 10 | | | * | * | | | | |
| 11 | | | ** | ** | * | | * | |
| 12 | ** | | | ** | | | * | |
| 13 | | * | ** | * | | | * | * |
| 14 | **† | ††††† | ††† | † | *†††††<br>†††††† | ††††††† | *††††† | *†††††† |
| Tc tals | 7 | 8 | 10 | 11 | 13 | 12 | 10 | 9 |

* Died during experiment.
† Killed at 7 months or at end of experiment (14 months).
A: no anti-diabetic treatment.
B: low-carbohydrate diet.
C: insulin treatment.
D: insulin and low-carbohydrate diet.

FIG. 1.—Epithelial renal tumour. H. and E. ×300.

FIG. 2.—Wholly mesenchymal renal tumour consisting of irregularly disposed round or spindle cells with irregular dark nuclei. H. and E. ×300.

*Wholly or predominantly mesenchymal tumours*

Thirty-two mesenchymal tumours were found varying in size from 2 to 15 cm. They were uniformly pale and mostly solid in appearance, but with some cystic areas. Microscopically, these tumours could be divided into two categories.

In the first category there were 8 wholly mesenchymal tumours consisting of sheets of loosely disposed undifferentiated round or spindle cells. The nuclei were large and hyperchromatic and mitoses were frequent. Little cytoplasm was present and the cell outline was faint (Fig. 2). At the edge of the tumours there was invasion of normal renal substance.

The second category consisted of 24 tumours similar to the 8 wholly mesenchymal tumours, except that they also contained epithelial elements. They were bilateral in 2 cases. Six animals had separate purely epithelial tumours, in addition to the predominantly mesenchymal tumours. Microscopically, in addition to sheets of spindle cells, there were ill-defined hypercellular areas composed of cells with hyperchromatic nuclei and little cytoplasm (Fig. 3). Pleomorphism and mitoses were common in both these patterns of mesenchymal growth. In most of the tumours, the connective-tissue elements showed fibrous differentiation only, but some contained smooth muscle, and in one case there were areas of cytologically malignant cartilage. The epithelial element was present in the form of tubules. Some tubules were widely dilated and contained papillary infoldings lined by flattened cells. Other tubules were small and lined by multilayered plump columnar cells often showing pleomorphism and an increased nuclear-cytoplasmic ratio but few mitoses (Fig. 4). Such tubules were frequently surrounded by condensations of stromal cells reminiscent of the human nephro-

FIG. 3.—Predominantly mesenchymal tumour with ill-defined hypercellular islands. H. and E. ×75.

FIG. 4.—Neoplastic tubules in a predominantly mesenchymal tumour. H. and E. ×185.

blastoma (Fig. 5) and in some there appeared to be a transition between neoplastic stromal cells and cells lining the tubular structures. At the edges of the tumour, non-neoplastic tubules were caught up in the advancing spindle-cell areas but centrally, the tubules with a stratified lining appeared to be truly neoplastic.

The distribution of the tumours showed no significant differences between the 4 treatment groups (Table II) but the tumours in the insulin-treated animals were associated with a higher mortality (Table I). Thus, there were 16 deaths out of 21 rats with tumours in Groups C and D compared with 7 out of 15 in Groups A and B ($\chi^2 = 4{\cdot}24$, $P < 0{\cdot}05$).

TABLE II.—*Distribution of Tumours and Fate of Rats in the Different Treatment Groups*

| Group | Died with tumour | Tumour found at end of experiment | Total animals with tumours | Tumour type: Epithelial | Tumour type: Wholly mesenchymal | Tumour type: Partly mesenchymal | Total tumours in each group |
|---|---|---|---|---|---|---|---|
| A | 5 | 2 | 7 | 3 | 2 | 3 | 8 |
| B | 2 | 6 | 8 | 5 | 2 | 6 | 13 |
| C | 6 | 4 | 10 | 2 | 2 | 7 | 11 |
| D | 10 | 1 | 11 | 4 | 2 | 8 | 14 |
| Totals | 23 | 13 | 36 | 14 | 8 | 24 | 46 |

A: no anti-diabetic treatment.
B: low-carbohydrate diet.
C: insulin treatment.
D: insulin and low-carbohydrate diet.
Each group consisted of 20 animals.

FIG. 5.—Epithelial structure in a hypercellular island of a predominantly mesenchymal tumour. H. and E. × 185.

DISCUSSION

To our knowledge, this is the first description of mesenchymal renal tumours developing in streptozotocin-treated rats, although the induction of epithelial renal tumours by streptozotocin is well documented (Arison and Feudale, 1967; Rakieten *et al.*, 1968; Mauer *et al.*, 1974*a*, *b*; Bennington and Beckwith, 1975) as is the induction of both types of tumour by other nitrosamine compounds (Magee and Barnes, 1959, 1962; Riopelle and Jasmin, 1969; Hard and Butler, 1970*a*, 1971).

The epithelial tumours are similar to those seen in man, where the distinction between adenoma and carcinoma is often made on the basis of size alone, the morphological and histochemical features being identical (Ericsson, Seljelid and Orrenius, 1966; Hard and Butler, 1970*b*; Fisher and Horvat, 1972). The pathogenesis of these epithelial tumours has been extensively studied by light and electron microscopy in rats given dimethylnitrosamine (Hard and Butler, 1971). They arise from proximal tubular cells, the earliest proliferative lesions being seen 6 weeks after the initial injury, which consists of sporadic areas of cell death and macrophage infiltration. Spontaneous familial renal adenomas of dominant inheritance have been recorded in Wistar rats (Eker and Mossige, 1961), but none of the control animals in this experiment developed such tumours.

The mesenchymal tumours seen in our animals appear to be malignant, as assessed by their microscopical appearances and evidence of local invasion, although again metastases were not found. These lesions have similar light microscopical characteristics to those induced in rat kidneys by dimethylnitrosamine (Hard and Butler, 1970*a*) and nitrosomethyl urea (Thomas, Wessel and Citoler, 1972). The pathogenesis of these tumours has also been intensively studied (Hard and Butler, 1971) and they appear to arise from proliferating mesenchymal cells in the juxtaglomerular region. In contrast to earlier workers (Magee and Barnes, 1959, 1962; Argus and Hoch Ligeti, 1961; Yang, 1966) and despite the well recognized mixed nature of human nephroblastomas, Hard and Butler do not accept that any of the rat neoplasms are true mixed tumours. The epithelial structures are all regarded by these workers as hyperplastic reactive tubules entrapped by the tumour, and this is supported by autoradiographic and histochemical studies (Thomas *et al.*, 1972). It is, therefore, advocated that such tumours produced by N-nitroso compounds should not be classified as nephroblastomas at this stage and that the term "stromal nephroma" should be used (Riopelle and Jasmin, 1969; Bennington and Beckwith, 1975). In several of the tumours in our animals, the microscopic features strongly suggested that the tubular structures were truly malignant, but we recognize that in the absence of metastases this has not been conclusively established. In

TABLE III.—*Streptozotocin-induced Renal Tumours in Rats: Comparison of Experimental Conditions*

| Workers | Strain of rat | Sex | Weight (g) | Dose of streptozotocin and route | Duration of experiment (months) | Tumours first detected (months) | Number of experimental animals | Animals developing tumour % | Type of tumour |
|---|---|---|---|---|---|---|---|---|---|
| Rakieten *et al.* | Sherman<br>Holtzman | M and F | M101–177<br>F92–154 | 50 mg/kg i.v. alone or with Zedalan | 16 | 5 | 38<br>75 | 29<br>16 | Epithelial (adenoma) |
| Mauer *et al.* | Sprague–Dawley<br>Lewis | M and F | 100 | 65 mg/kg i.v. | 16 | 3 | 60<br>100 | 18<br>16 | Epithelial (adeno-carcinoma) |
| Arison & Feudale | Holtzman | M | Adult | 50 mg/kg i.v. | 16 | 8 | — | 53 | Epithelial (potentially malignant) |
| †Magnall & Slater | PVG/C | M | 200 | 65 mg/kg i.p. | 13 | — | 18 | nil | — |
| Present series | Wistar (Cummings–Sprague–Europe) | M | 300 | 25 mg/kg i.v. | 14 | 7 | 80 | 16<br>26 | Epithelial<br>Mesenchymal |

† Personal communication (1977), for which we are grateful.

comparing tumours of the rat kidney to human nephroblastomas, note must be taken of evidence supporting the origin of nephroblastomas from persistent nests of metanephric blastoma which may be found in the kidneys of infants (Potter, 1972; Bove, Koffler and McAdams, 1969; Bennington and Beckwith, 1975; Shanklin and Sotelo-Avila, 1969). Such areas were not seen in the rat kidneys studied here and the tumours appear to arise from fully differentiated cells. Spontaneous nephroblastomas do occur rarely in Wistar rats (Pittermann, 1974) but these contain typical glomeruloid structures which were not seen in our tumours.

In all our animals the non-tumorous renal tissue showed varying degrees of interstitial nephritis. In man, epithelial neoplasms develop particularly in scarred kidneys and it is likely that interstitial nephritis has played a role in the development of such tumours in our experimental rats, but the relevance of interstitial nephritis to the development of mesenchymal tumours is uncertain.

The diabetic state does not appear to be causally related to the development of streptozotocin-induced epithelial tumours (Mauer *et al.*, 1974*b*). However, in our experiment, more rats died from their renal tumours in the insulin-treated groups, suggesting that metabolic status may influence the subsequent growth of the tumour.

Puzzling differences exist between different laboratories in the frequency and type of renal tumours in streptozotocin-treated rats. This diabetogenic agent is widely employed in research, but other workers have either not encountered any tumours, or only epithelial neoplasm (Table III). The epithelial tumours were first noted by other workers after 3–8 months. In our animals, these lesions were not noted until the animals died with large tumours from 11 months onwards. Twenty-six per cent of our animals developed mesenchymal tumours, which so far have not been reported by other workers. These tumours were first seen in animals killed at 7 months, and from then on in animals dying up to the end of the experiment. The strain of rat as well as the metabolic status may be important factors in the development of different tumour types.

We are grateful to the MRC for financial support, to Miss V. Wright for looking after the experimental animals, Mr R. Leaver and staff, Mr J. Fenton and Miss J. Conway for technical, photographic and secretarial assistance.

### REFERENCES

Argus, M. F. & Hoch Ligeti, C. (1961) Comparative Study of the Carcinogenic Activity of Nitrosamines. *J. natn. Cancer Inst.*, **27**, 695.

Arison, R. N. & Feudale, E. L. (1967) Induction of Renal Tumour by Streptozotocin in Rats. *Nature, Lond.*, **214**, 1254.

Bennington, J. L. & Beckwith, J. B. (1975) Tumours of the Kidney, Renal Pelvis and Ureter. In *Atlas of Tumour Pathology, 2nd Series, Fascicle 12.* Armed Forces Inst. Path., Washington, D.C.

Bove, K. E., Koffler, H. & McAdams, A. J. (1969) Nodular Renal Blastoma: Definition and Possible Significance. *Cancer, N.Y.*, **24**, 323.

Du Priest, R. W., Jr, Huntington, M. C., Massey, W. H., Weirs, A. J., Wilson, W. L. & Flether, W. S. (1975) Streptozotocin Therapy in 22 Cancer Patients. *Cancer, N.Y.*, **35**, 358.

Editorial (1975) Streptozotocin for Pancreatic Cholera. *Lancet*, i, 1327.

Eker, R. & Mossige, J. A. (1961) A Dominant Gene for Renal Adenomas in the Rat. *Nature, Lond.*, **189**, 858.

Ericsson, J. L. E., Seljelid, R. & Orrenius, S. (1966) Comparative Light and Electron Microscopic Observations of the Cytoplasmic Matrix in Renal Carcinomas. *Arch. Path. Anat. Physiol.*, **341**, 204.

Fisher, E. R. & Horvat, B. (1972) Comparative Ultrastructural Study of So-called Renal Adenoma and Carcinoma. *J. Urol.*, **108**, 382.

Fox, C., Ireland, J. T. & Sönksen, P. H. (1976) The Effect of Diabetic Control on Glomerular Basement Membrane Thickening. *Diabetologia*, **12**, 391.

Gagel, R. F., Costanza, M. E., De Lellis, R. A., Norton, R. A., Bloom, S. R., Miller, H. H., Angelo Ucci and Nathanson, L. (1976) Streptozotocin-treated Verner–Morrison Syndrome Plasma Vasoactive Intestinal Peptide and Tumour Responses. *Arch. Intern. Med.*, **136**, 1429.

Hard, G. C. & Butler, W. H. (1970*a*) Cellular Analysis of Renal Neoplasia: Induction of Renal Tumours in Dietary-conditioned Rats by Dimethyl-nitrosamine, with a Reappraisal of Morphological Characteristics. *Cancer Res.*, **30**, 2796.

Hard, G. C. & Butler, W. H. (1970*b*) Cellular Analysis of Renal Neoplasm: Light Microscopic Study of the Development of Interstitial Lesions Induced in the Rat Kidney by a Single Carcinogenic Dose of Dimethylnitrosamine. *Cancer Res.*, **30**, 2806.

Hard, G. C. & Butler, W. H. (1971) Morphogenesis of Epithelial Neoplasms Induced in the Rat Kidney by Dimethylnitrosamine. *Cancer Res.*, **31**, 1496.

Herr, R. R., Eble, T. E., Bergy, M. E. & Jahnke, H. K. (1960) *Antibiot. Ann.*, 236. (New York Medical Encyclopedia Inc.),

Jurod, A., Lambert, A. E., Orci, L., Putet, R., Goret, A. E. & Renold, A. E. (1967) Studies of the Diabetogenic Action of Streptozotocin. *Proc. Soc. exp. Biol. Med.*, **126**, 201.

Karunanayake, E. H., Baker, J. R. J., Christian, R. A., Hearse, D. J. & Mellows, G. (1976) Autoradiographic Study of the Distribution and Cell Uptake of $^{14}C$ Streptozotocin in the Rat. *Diabetologia*, **12**, 123.

Magee, P. N. & Barnes, J. M. (1959) The Experimental Production of Tumours in the Rat by Dimethylnitrosamine (N-Nitroso Dimethylamine). *Acta. Un. int. Cancr.*, **15**, 187.

Magee, P. N. & Barnes, J. M. (1962) Induction of Kidney Tumours in the Rat with Dimethylnitrosamine (N-Nitrosodimethylamine). *J. Path. Bact.*, **84**, 19.

Mauer, S. M., Sutherland, D. E. R., Steffes, M. W., Lee, C. S., Nafarian, J. S. & Brown, D. M. (1974*a*) Effects of Kidney and Pancreas Transplantation on Streptozotocin-induced Malignant Kidney Tumours in Rats. *Cancer Res.*, **34**, 1643.

Mauer, S. M., Lee, C. S., Nafarian, J. S. & Brown, D. M. (1974*b*) Induction of Malignant Kidney Tumours in Rats with Streptozotocin. *Cancer Res.*, **34**, 158.

Murray-Lyon, I. M., Eddleston, A. L. W. F., Williams, R., Brown, M., Itogbin, B. M., Bennett, A., Edwards, J. C. & Taylor, K. W. (1968) Treatment of Multiple-hormone-producing Malignant Islet-cell Tumour with Streptozotocin. *Lancet*, ii, 895.

Pittermann, W. (1976) In *Wilm's Tumour.* Ed. Pochedly, C. and Miller, D. Wiley Biomed. Publ., John Wiley and Sons Inc., p. 84.

Potter, E. L. (1972) *Normal and Abnormal Development of the Kidney.* Chicago: Year Book Medical Publishers.

Rakieten, N., Rakieten, M. L. & Nadkarni, M. V. (1963). *Cancer Chemother. Rep.*, **29**, 91.

Rakieten, N., Gordon, B. S., Cooney, D. A., Davis, R. D. & Schein, P. S. (1968) Renal Tumorigenic Action of Streptozotocin (NSC–85998) in Rats. *Cancer Chemother. Rep.*, **52**, 563.

Riopelle, J. L. & Jasmin, G. (1969) Nature, Classification and Nomenclature of Kidney Tumours Induced in the Rat by Dimethylnitrosamine. *J. natn. Cancer Inst.*, **42**, 643.

Schein, P. S., O'Connell, M. J., Blom, J., Hubbard, S., Magrath, I. T., Bergevin, P., Wiernik, P. H., Ziegler, J. L. & DeVita, V. T. (1974) Clinicl Antitumour Activity of Streptozotocin (NSC–85998). *Cancer, N.Y.*, **34**, 993.

Shanklin, D. R. & Sotelo-Avila, C. (1969) *In situ* Tumours in Fetuses, Newborns and Young Infants. *Biol. Neonate*, **14**, 286.

Thomas, C., Wessel, W. & Citoler, R. (1972) Histochemische elektronenmikroskopische und autoradiographische Untersuchungen an experimentell erzeugten Nephroblastomen. *Beitr. Path.*, **145**, 68.

Yang, Y. H. (1966) Renal Hyperplasia in Rats given Dimethylnitrosamine. *Urol. inter.*, **21**, 229.